# MUC1 Protects Preimplantation Embryos In Vitro via Clearance of ROS by Triggering Mitophagy

**DOI:** 10.3390/cells15090806

**Published:** 2026-04-29

**Authors:** Jingping Yang, Danjun Li, Chihyu Yang, Huayun Deng, Kaibo Lin, Bing Liao, Xiaodong Liao, Yue Liu, Qifeng Lyu, Lei Huang

**Affiliations:** 1 Department of Histoembryology, Genetics and Developmental Biology, Key Laboratory of Cell Differentiation and Apoptosis of Chinese Ministry of Education, Shanghai Key Laboratory of Reproductive Medicine, Shanghai Jiao Tong University School of Medicine, Shanghai 201318, China; jp_yang01@163.com (J.Y.); 15000233635@163.com (C.Y.); denghuayun0796@shsmu.edu.cn (H.D.); liaobing@shsmu.edu.cn (B.L.); liaoxd67@hotmail.com (X.L.);; 2 Department of Assisted Reproduction, Shanghai Ninth People’s Hospital, Shanghai Jiao Tong University School of Medicine, Shanghai 200011, China

**Keywords:** MUC1, embryos, mitophagy, ROS, preimplantation

## Abstract

Embryos being treated using assisted reproductive technology (ART) are unavoidably exposed to physical stressors, thus producing reactive oxygen species (ROS) which trigger mitophagy to support embryonic development. However, the mechanisms underlying the regulation of mitophagy in early embryonic development remain largely unexplored. Here, we found that Mucin 1 (MUC1) exhibited a uniform distribution in both mouse and human oocytes, and its expression peaked at the blastocyst stage. Further analysis revealed that *Muc1* knockout impairs blastocyst formation in vitro. Correspondingly, *Muc1* knockout led to the accumulation of mitochondrial reactive oxygen species (mtROS) and a reduction in phosphatase and tensin homolog (PTEN)-induced putative kinase 1 (PINK1)/Parkinson protein 2 (PARK2/Parkin)-dependent mitophagy. Stimulation of mitophagy via low-dose carbonyl cyanide 3-chlorophenylhydrazone (CCCP) treatment rescued the blastocyst formation defect in *Muc1*-null embryos. Vitamin C supplementation effectively scavenged mtROS and restored developmental competence. Together, our findings establish that MUC1 safeguards early embryonic development by promoting mitophagy to decrease mtROS levels in vitro. Moreover, vitamin C could compensate for *Muc1* deficiency by eliminating mtROS. This study not only identified a new function of MUC1 in protecting early embryonic development in vitro, but also revealed a novel mechanism of mitophagy regulation in early embryos, which has potential applications for ART.

## 1. Introduction

It is estimated that 143 million women and 55 million men were affected by infertility worldwide in 2021 [1]. Assisted reproductive technology (ART) has emerged as a key solution to infertility [2,3,4], but the total fertility rate (TFR) remains lower than 3% in many countries [5]. Embryos cultured in vitro are unavoidably exposed to physical stressors, including fluctuations in temperature, pH, oxygen tension, humidity, and electromagnetic radiation [6], which leads to the generation of reactive oxygen species (ROS) and negative effects on embryonic development [7]. Thus, exploring the factors that influence in vitro embryonic development and identifying strategies to optimize culture conditions are key long-term research objectives.

Mitophagy is a selective process that is responsible for the labeling, elimination, and recycling of damaged and redundant mitochondria [8]. In response to excessive ROS production, mitophagy is activated to remove damaged mitochondria, thereby reducing mitochondrial ROS (mtROS) and maintaining cellular homeostasis [9]. In contrast, impaired mitophagy causes the accumulation of mtROS and damage to mitochondria, ultimately leading to cellular injury or even cell death [10]. Under oxidative stress, supplementation of porcine oocytes with melatonin has been shown to upregulate mitophagy-related markers and reduce ROS levels [11]. In aged mouse oocytes, blocking the conversion of cholesterol to cholesterol esters decreases mitophagy activity, induces mitochondrial dysfunction, and leads to excessive ROS accumulation [12]. Gestational exposure to black phosphorus nanoparticles has been reported to elevate embryonic ROS levels, activate mitophagy, and induce placental trophoblast dysfunction, which culminates in fetal developmental impairment [13]. The phosphatase and tensin homolog (PTEN)-induced putative kinase 1 (PINK1)/Parkinson protein 2 (PARK2/Parkin)-dependent pathway is the best characterized mitophagy pathway. In early embryonic development, paternal mitochondria elimination is mitophagy-dependent, thereby ensuring maternal inheritance of mitochondrial DNA (mtDNA) [14,15]. Spent embryo culture medium from infertile patients has been shown to exhibit reduced PINK1 transcript levels compared with normal controls [16]. Copper oxide nanoparticles impair mouse preimplantation embryonic development by disrupting mitophagy-mediated metabolism [17]. Lysine-specific demethylase 1 (LSD1) coordinates mitophagy and other physiological processes to gate porcine early embryonic development [18]. By maintaining cellular energy metabolism and oxidative stress homeostasis, mitophagy presumably provides sufficient energy support for blastocyst cell migration, embryo adhesion, and embryonic development [19]; however, the underlying mechanisms remain largely unknown.

Mucin 1 (MUC1) is the most well-known transmembrane protein in the mucin family, which features a highly glycosylated extracellular domain. Under normal conditions, MUC1 is predominantly localized on the surface of epithelial cells, exerting protective, lubricating, and hydrating functions [20]. MUC1 has been reported to localize on uterine epithelial cells and is downregulated during embryo implantation, permitting embryo attachment to the uterus [21]. Despite this, the expression of MUC1 during the peri-implantation period remains indispensable. A clinical study has shown that patients with unexplained recurrent pregnancy loss exhibit significantly decreased MUC1 expression in the endometrium compared to the control group [22]. Trophoblast cells isolated from placentas display strong MUC1 staining [23]. When human embryos are co-cultured with endometrial epithelial monolayers, MUC1 expression is depleted in epithelial cells at and adjacent to the sites of embryo attachment while remaining intact in neighboring cells [24]. However, the function of MUC1 in early embryonic development requires further clarification. Our previous work reported that MUC1 promotes mitophagy through protecting PINK1 from ATPase family AAA domain-containing 3A (ATAD3A)-mediated proteolysis in breast carcinoma cells [25]. Based on these findings, we hypothesize that MUC1 influences early embryonic development by regulating mitophagy.

Here, we found that *Muc1* deletion leads to elevated mtROS levels and reduced mitophagy activity, impairing early embryonic development in vitro. Notably, this study discovered a novel protective role of MUC1 during in vitro early embryonic development and elucidated a unique regulatory mechanism for mitophagy in early embryos.

## 2. Materials and Methods

### 2.1. Human Oocytes and Spermatozoa

The human oocytes and spermatozoa used in this study were donated for research with informed consent provided by patients. This study was approved by the Ethics Committee of the Ninth People’s Hospital, Shanghai Jiao Tong University School of Medicine (Approval No. 20181101).

### 2.2. Animal Studies

All animal procedures were performed in accordance with the Shanghai Medical Experimental Animal Care Guidelines and were approved by the Institutional Animal Care and Use Committee of Shanghai Jiao Tong University School of Medicine (Approval No. JUMC2023-150-A). Wild-type and *Muc1* knockout mice, both on the C57BL/6J background, were generated by the Shanghai Model Organisms Center, Inc. (Shanghai, China), as previously described [26]. The mice were housed under specific pathogen-free conditions in a controlled environment (20~22 °C, 50~70% humidity) with a 12-h light/dark cycle. Genotyping was performed via PCR using the primers listed in Table 1.

### 2.3. Drugs and Antibodies

The following drugs and antibodies were used in this study: carbonyl cyanide 3-chlorophenylhydrazone (CCCP; MedChemExpress, Monmouth Junction, NJ, USA), vitamin C (Sigma-Aldrich, Aachen, Germany), monoclonal rabbit anti-MUC1 (Abcam, Cambridge, UK), monoclonal mouse anti-octamer-binding transcription factor 3/4 (Oct-3/4) (Santa Cruz, Dallas, TX, USA), monoclonal rabbit anti-caudal type homeobox 2 (CDX2) (Abcam, Cambridge, UK), polyclonal rabbit anti-LC3 (Proteintech, Wuhan, China), monoclonal rabbit anti-sequestosome 1 (SQSTM1/p62) (Selleck, Houston, TX, USA), polyclonal rabbit anti-PINK1 (Proteintech, Wuhan, China), polyclonal rabbit anti-Parkin (Proteintech, Wuhan, China), donkey anti-rabbit Alexa Fluor™ 488/555, and donkey anti-mouse Alexa Fluor™ 488 (Invitrogen, Carlsbad, CA, USA).

### 2.4. In Vitro Fertilization (IVF)

IVF was performed following an established protocol with minor modifications [27]. Briefly, female mice aged 4–6 weeks were hormonally primed by intraperitoneal administration of 10 IU equine chorionic gonadotropin (eCG; Ningbo No. 2 Hormone Factory, Ningbo, China), followed 48 h later by administration of 10 IU human chorionic gonadotrophin (hCG; Ningbo No. 2 Hormone Factory, Ningbo, China). Spermatozoa were harvested from the cauda epididymides of 10~12-week-old male mice and capacitated in droplets of Human Tubal Fluid (HTF; Merck, Darmstadt, Germany). Cumulus–oocyte complexes (COCs) retrieved from superovulated females were subsequently added to the HTF medium containing spermatozoa. Following co-incubation for 5–6 h, presumptive zygotes were thoroughly rinsed in HTF, then transferred to potassium simplex optimization medium (KSOM; Merck, Darmstadt, Germany) and cultured at 37 °C in a 5% CO_2_, 5% O_2_ atmosphere. Embryonic development was monitored at embryonic days 0.5 (D0.5), 1.5 (D1.5), 2.5 (D2.5), 3.5 (D3.5), and 4.5 (D4.5).

### 2.5. Fertility Test

Ten-week-old male C57BL/6J mice were paired with individual 6-week-old females of the same strain for continuous breeding over a 6-month period. Breeding pairs were established using mice with identical genotypes (wild-type × wild-type or *Muc1* knockout × *Muc1* knockout), with six pairs per genotype group. The total number of pups, number of surviving offspring and sex ratio were recorded for each parturition, along with the delivery date. These parameters were used to calculate litter size, survival rate of pups, sex ratio, and interpregnancy interval, which are core indicators of reproductive capacity.

### 2.6. Live-Cell Staining

To assess mtROS levels, embryos were collected and incubated for 30 min at 37 °C in a humidified atmosphere containing 5% CO_2_ in Hank’s balanced salt solution (HBSS) supplemented with bovine serum albumin (60 µg/mL), polyvinylpyrrolidone (1 mg/mL), MitoSOX Red (5 µM; Invitrogen, Carlsbad, CA, USA), and Hoechst 33342 (1 µg/mL; Beyotime, Shanghai, China). Following incubation, the embryos were washed three times and mounted in pre-warmed HBSS for confocal imaging using a confocal microscope (Olympus, Tokyo, Japan).

To measure the mitochondrial membrane potential, embryos were incubated in KSOM supplemented with JC-1 Dye (12.5 µg/mL; Beyotime, Shanghai, China) for 20 min at 37 °C. After washing in KSOM three times, the embryos were observed under a laser confocal microscope as described above.

To assess the co-localization of mitochondria and lysosomes, blastocysts were incubated at 37 °C for 30 min in a pre-warmed HBSS-based solution containing LysoTracker (200 nM; Beyotime, Shanghai, China) and MitoTracker (200 nM; Beyotime, Shanghai, China). The embryos were subsequently incubated for an additional 15 min at 37 °C in HBSS supplemented with Hoechst 33342. Following three washes with HBSS, the samples were subjected to confocal imaging, as described above.

To assess the co-localization of mitochondria and autophagosomes, mitochondrial labeling was carried out in live embryos prior to fixation. Embryos were incubated at 37 °C for 30 min in pre-warmed phosphate-buffered saline (PBS) supplemented with MitoTracker Red CMXRos (500 nM; Invitrogen, Carlsbad, CA, USA), followed by three washes with PBS. Samples were then fixed in pre-warmed 4% paraformaldehyde (PFA) at 37 °C for 15 min and processed for LC3 staining.

### 2.7. qPCR for Telomere Measurement

A single-cell telomere length (TL) assay was used following a published protocol [28]. In brief, 2 µL of 2 × lysis buffer was added to a 0.2 mL PCR tube containing a single embryo, and the mixture was incubated at 95 °C for 10 min. The 2 × lysis buffer is composed of 100 mM pH 7.4 2-amino-2-(hydroxymethyl)-1,3-propanediol (Tris)·HCl, 300 mM NaCl, 0.8 mM ethylenediaminetetraacetic acid (EDTA), 2% Nonidet P 40 (NP-40), and 5 mM DL-dithiothreitol (DTT). Pre-PCR amplification was carried out using Phanta Flash Super-Fidelity DNA Polymerase (Vazyme, Nanjing, China). The reaction was prepared as follows: 2 µL single-cell genomic DNA, 10 µL 2 × Phanta Flash Master Mix, 1 µL each of forward and reverse telomere primers (10 µM), 1 µL each of forward and reverse reference gene primers (10 µM), and 6 µL of ddH_2_O. The thermal cycling conditions were as follows: initial denaturation at 98 °C for 30 s, followed by 16 cycles of denaturation at 98 °C for 10 s, annealing at 60 °C for 5 s, and extension at 72 °C for 10 s, with a final extension at 72 °C for 1 min. The PCR products were purified using a purification kit (Zymo Research, Irvine, CA, USA) and eluted in 100 µL of ddH_2_O.

DNA concentration was measured using a NanoDrop spectrophotometer (Thermo Fisher Scientific, Waltham, MA, USA) and normalized to 4 ng/µL using 10-fold diluted working aliquots. Quantitative PCR (qPCR) was performed using a Bio-Rad thermocycler (Thermo Fisher Scientific, Waltham, MA, USA) using the same primers that were used for pre-PCR amplification. The reaction conditions were as follows: initial denaturation at 95 °C for 10 min, followed by 40 cycles of amplification (95 °C for 15 s, 60 °C for 30 s, and 72 °C for 30 s), and 80 cycles of melting curve analysis (60 °C to 95 °C). The relative TL for each single cell was calculated using the ΔΔCt method, comparing the telomere (T) and reference gene (R) values. The relative mean TL was determined by qPCR. The primer sequences are provided in Table 1.

### 2.8. mRNA Extraction and cDNA Generation

For ovarian tissue samples, total RNA was extracted using TRIzol reagent. Following normalization of RNA concentrations, cDNA was synthesized via reverse transcription using HiScript III Reverse Transcriptase (Vazyme, Nanjing, China), and the resulting cDNA was used for qPCR analysis.

Embryos were individually collected and processed using a single-cell sequence-specific amplification kit (Vazyme, Nanjing, China). Reverse transcription and cDNA pre-amplification were performed at 50 °C for 60 min, followed by denaturation at 95 °C for 3 min and 18 cycles of amplification (95 °C for 15 s and 60 °C for 15 min), with a final hold at 4 °C. Amplified cDNA was diluted at a 1:10 ratio and used as the template for quantitative PCR.

### 2.9. RT-qPCR

qPCR was carried out using ChamQ Universal SYBR qPCR Master Mix (Vazyme, Nanjing, China) and a QuantStudio 6-Flex Real-Time PCR System (Thermo Fisher Scientific, Waltham, MA, USA), according to the manufacturer’s instructions. β-actin was used as the internal reference gene. The primer sequences are provided in Table 1. All assays were performed in triplicate, and relative gene expression was calculated using the ΔΔCt method.

### 2.10. Immunofluorescence Staining

The samples were fixed with 4% PFA at room temperature for 1 h, followed by three washes with washing buffer (PBS solution containing 1% bovine serum albumin (BSA; Bio Basic Inc., Hong Kong, China)). Subsequently, the samples were permeabilized with 0.25% Triton X-100 for 20 min. After washing, blocking was performed using 1% BSA in PBS for 1 h. Following incubation with primary antibody overnight at 4 °C, the samples were washed and then incubated with secondary antibody for 1 h at room temperature. Subsequently, nuclei were stained with 4′,6-diamidino-2-phenylindole (DAPI, Vector Laboratories, Burlingame, CA, USA) for 15 min. Following three washes, the embryos were mounted in 1% BSA for confocal imaging, as described above. Images were acquired under identical settings to ensure comparability across samples. Fluorescence intensity was quantified with ImageJ software (version 1.54k; National Institutes of Health, Bethesda, MD, USA) and normalized by the background-subtracted mean fluorescence intensity method. Quantification of LC3 puncta was performed following this reference [29].

### 2.11. Statistical Analysis

All data were tested for normality using the Shapiro–Wilk test. Statistical analyses were performed using the SPSS software (version 28.0; IBM, Armonk,, NY, USA). Comparisons between two groups were conducted using two-tailed Student’s *t*-test or Chi-square test, while multiple-group comparisons were analyzed using one-way analysis of variance (ANOVA). All figures were plotted with the GraphPad Prism software (version 10.4; GraphPad Software, San Diego, CA, USA). Differences were considered statistically significant if *p* < 0.05.

## 3. Results

### 3.1. Expression and Localization of MUC1 in Gametes and Preimplantation Embryos

**Figure 1:**
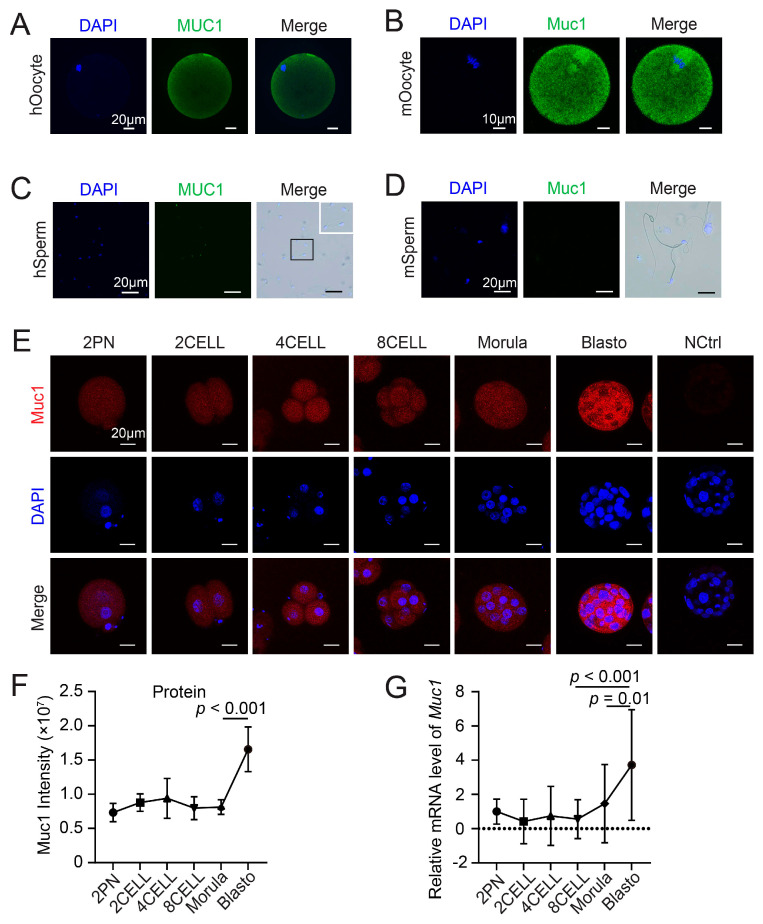
Expression and localization of MUC1 in gametes and preimplantation embryos. (**A**–**D**) Representative immunofluorescence images stained with antibody against MUC1 (green) and co-stained with DAPI (to stain DNA, blue) in human oocyte, mouse oocyte, human sperm, and mouse sperm, respectively. Magnified immunofluorescence image of human sperm is presented within the white box in (**C**). (**E**) Representative images stained with antibody against MUC1 (red) and co-stained with DAPI (to stain DNA, blue) from the 2PN stage to the blastocyst stage. NCtrl, negative control (no primary antibody). Scale bars: 20 µm. (**F**) Quantification of MUC1 fluorescence intensity using ImageJ. Number of samples analyzed at each stage: 2PN = 23, 2CELL = 25, 4CELL = 23, 8CELL = 23, MORULA = 19, and BLASTO = 29 (analyzed using ANOVA). (**G**) Real-time quantitative PCR results for *Muc1* mRNA expression levels during early mouse embryonic development. Relative expression level of *Muc1* is presented as fold change normalized to β-ACTIN. Number of samples analyzed at each stage: 2PN = 20, 2CELL = 16, 4CELL = 23, 8CELL = 17, MORULA = 17, and BLASTO = 15 (analyzed using ANOVA). Data in (**F**,**G**) are means ± SDs from at least three independent experiments.

To determine whether MUC1 is involved in embryogenesis, immunofluorescence (IF) staining was performed on mouse and human gametes. The results showed that MUC1 exhibited a uniform distribution in both mouse and human oocytes (Figure 1A,B). Interestingly, MUC1 was localized exclusively to the neck region of human sperm (Figure 1C), whereas little expression of MUC1 was detected in mouse sperm (Figure 1D). Next, the expression of MUC1 during mouse preimplantation development was examined. The IF results showed that MUC1 was predominantly localized in the cytoplasm throughout preimplantation development (Figure 1E). The intensity of MUC1 fluorescence revealed a modest increase at the 4-cell stage and a progressive accumulation from the morula stage to the blastocyst stage, with peak protein levels occurring at the blastocyst stage (Figure 1F). Consistent with the protein levels, RT-qPCR analysis demonstrated that *Muc1* transcripts were maintained at relatively low abundance from the pronuclear (2PN) stage to the 8-cell stage, with a significant increase from the morula stage to the blastocyst stage (Figure 1G). These data indicate the presence of MUC1 in gametes and mouse embryos, suggesting that MUC1 may play a role in early embryonic development.

### 3.2. Muc1 Knockout Has Little, if Any, Effect on Fertility in Mice

**Figure 2:**
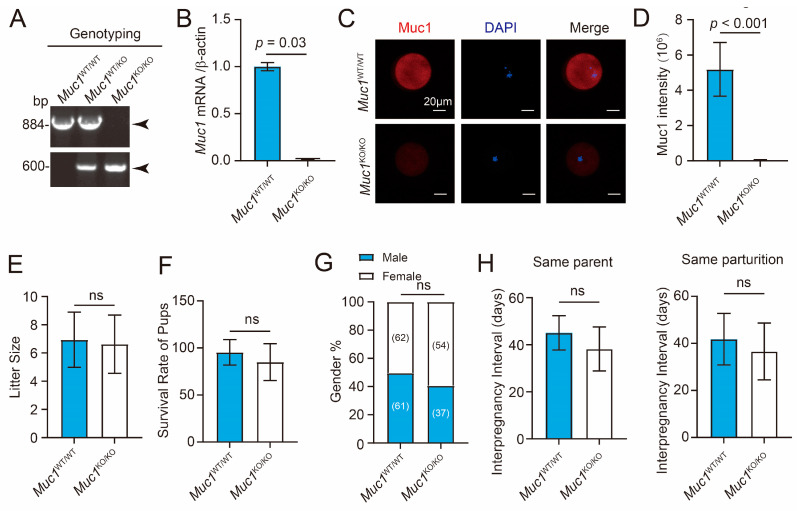
*Muc1* knockout has little, if any, effect on fertility in mice. (**A**) Genotyping of wild-type and *Muc1* knockout mice. The black arrows indicate the target bands. (**B**) RT-qPCR analysis of wild-type and *Muc1* knockout ovarian tissues. Relative expression level of *Muc1* is presented as fold change normalized to β-ACTIN (analyzed using two-tailed Student’s *t*-test). (**C**) Representative immunofluorescence images depicting MUC1 expression in MII oocytes of wild-type and *Muc1* knockout mice. MII, metaphase II. Scale bar: 20 µm. (**D**) Quantification of relative MUC1 fluorescence intensity in MII oocytes of wild-type and *Muc1* knockout mice. Number of samples: wild-type = 33 and *Muc1* knockout = 28 (analyzed using two-tailed Student’s *t*-test). (**E**) Litter size of wild-type and *Muc1* knockout pairs (analyzed using two-tailed Student’s *t*-test). (**F**) Survival rate of pups of wild-type and *Muc1* knockout pairs (analyzed using two-tailed Student’s *t*-test). (**G**) Offspring sex ratio from wild-type and *Muc1* knockout pairs (analyzed using Chi-square test). (**H**) Interpregnancy interval of wild-type and *Muc1* knockout pairs for same parents (**left**) and parturition (**right**) (analyzed using two-tailed Student’s *t*-test). Data in (**B**,**D**) are means ± SDs from at least three independent experiments. ns: not significant.

To assess the role of MUC1 in mouse embryonic development, the *Muc1* global knockout mouse model was utilized [26]. The *Muc1* deficiency was initially confirmed by genotyping (Figure 2A), indicating the successful knockout of *Muc1*. RT-qPCR analysis of *Muc1* in ovarian tissues further revealed a significant reduction in *Muc1* mRNA levels (Figure 2B). Moreover, immunofluorescence staining demonstrated a marked decrease in MUC1 levels in metaphase II (MII) oocytes (Figure 2C,D). To assess whether loss of *Muc1* impacts embryonic development under physiological conditions, we examined multiple reproductive parameters, including litter size, neonatal survival, offspring sex ratio, and interbirth interval. Genotype-matched females and males (wild-type pairs or *Muc1* knockout pairs) were housed for continuous breeding at a 1:1 ratio, with six independent breeding units per genotype. Reproductive outcomes were recorded over a six-month period. No significant differences were observed in litter size or survival rate of offspring (Figure 2E,F), although there was a decreasing trend for offspring survival rate. The offspring sex distribution remained indistinguishable between wild-type and *Muc1* knockout genotypes across all reproductive cycles (Figure 2G). In addition, analysis of the interpregnancy interval revealed no obvious difference between the wild-type and *Muc1* knockout groups (Figure 2H). Collectively, these findings demonstrate that *Muc1* deficiency has no effect on fertility in mice.

### 3.3. Muc1 Knockout Impairs In Vitro Embryonic Development in Mice

**Figure 3:**
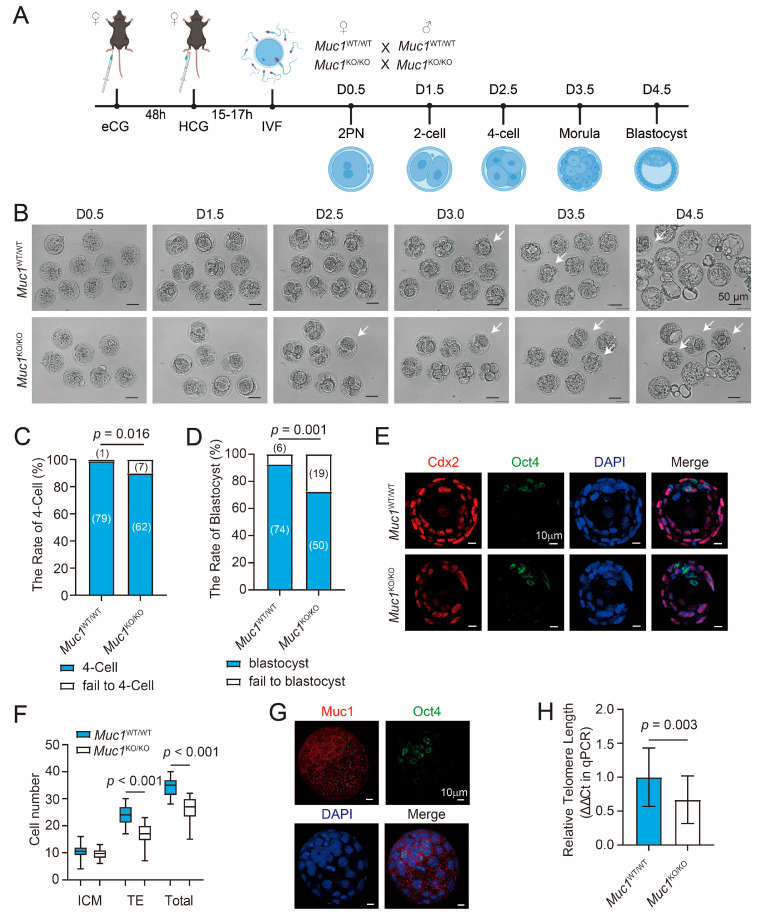
*Muc1* knockout impairs in vitro embryonic development in mice. (**A**) Overview of the experimental design (created in BioRender; https://BioRender.com/2uu2xma (accessed on 25th February 2026)). (**B**) Morphological comparison of D0.5, D1.5, D2.5, D3.0, D3.5, and D4.5 wild-type and *Muc1* knockout embryos. Scale bar: 50 µm. White arrows indicate embryos with developmental arrest. (**C**) The rate of embryos that reached the 4-cell stage in wild-type and *Muc1* knockout groups. Number of samples: wild-type = 80 and *Muc1* knockout = 69 (analyzed using Chi-square test). (**D**) The rate of embryos that reached the blastocyst stage in wild-type and *Muc1* knockout groups. Number of samples: wild-type = 80 and *Muc1* knockout = 69 (analyzed using Chi-square test). (**E**) Representative immunofluorescence images stained with antibodies against Oct4 (green, ICM) and CDX2 (red, TE), and co-stained with DAPI (blue, nuclei). Scale bar: 10 µm. (**F**) The number of cells in blastocysts (ICM, TE, or total) of wild-type and *Muc1* knockout groups based on three-dimensional reconstruction. Number of samples: wild-type = 20 and *Muc1* knockout = 20 (analyzed using two-tailed Student’s *t*-test). (**G**) Representative immunofluorescence images stained with antibodies against Oct4 (green) and MUC1 (red), and co-stained with DAPI (blue). Scale bar: 10 µm. (**H**) Relative telomere length of wild-type and *Muc1* knockout blastocysts. Number of samples: wild-type = 27 and *Muc1* knockout = 27 (analyzed using two-tailed Student’s *t*-test). Data in (**C**,**D**,**F**,**H**) are means ± SDs from at least three independent experiments.

The process for exploring the effect of MUC1 on early embryonic development in vitro is depicted in Figure 3A. Wild-type and *Muc1* knockout females were hormonally super-ovulated, and IVF was performed to obtain genotype-matched zygotes, which were subsequently cultured in vitro to the blastocyst stage. Embryonic development was monitored daily. The results revealed that *Muc1* knockout embryos displayed a markedly reduced capacity to reach the blastocyst stage compared with the wild-type controls (Figure 3B). This reduction in 4-cell-stage and blastocyst-stage embryos was further confirmed by Chi-square analysis (Figure 3C,D), indicating compromised embryonic development following *Muc1* deletion. OCT4 is a key pluripotency marker that is specifically expressed in the inner cell mass (ICM) to maintain stemness, whereas CDX2 acts as a trophectoderm (TE)-specific transcription factor that drives trophoblast lineage differentiation [30]. To investigate whether MUC1affects blastocyst lineage allocation, immunofluorescence staining of OCT4 and CDX2 was performed in blastocysts (Figure 3E), followed by three-dimensional reconstruction-based counting (Figure 3F). The number of ICM cells (10.45 ± 2.86, *n*  =  20 vs. 9.70 ± 1.87, *n*  =  20, *p*  =  0.3318) was comparable between wild-type and *Muc1* knockout blastocysts, whereas both the number of TE cells (23.90 ± 3.63, *n*  =  20 vs. 16.80 ± 4.24, *n*  =  20, *p* < 0.0001) and the number of total cells (34.35 ± 3.45, *n*  =  20 vs. 26.40 ± 4.38, *n*  =  20, *p* < 0.0001) were significantly decreased in *Muc1* knockout blastocysts (Figure 3F). These results suggest that *Muc1* knockout preferentially impairs trophectoderm rather than ICM at the blastocyst stage. To figure out this phenomenon, we examined whether MUC1 expression was different in TE and ICM. Thus, co-staining for MUC1 and OCT4 was performed (Figure 3G). MUC1 expression was detected in both the ICM and TE, suggesting no expression bias to the TE. Given that cellular oxidative stress significantly contributes to telomere shortening [31], qPCR was performed to assess the relative telomere length (Figure 3H). Telomere length was significantly shorter in *Muc1* knockout blastocysts compared with wild-type blastocysts, suggesting reduced developmental potential following *Muc1* knockout. These results indicate that *Muc1* knockout compromises early embryonic development in vitro, impairing the trophectoderm and eroding the blastocyst developmental potential.

### 3.4. Muc1 Knockout Leads to Accumulation of mtROS and Damaged Mitochondria

**Figure 4:**
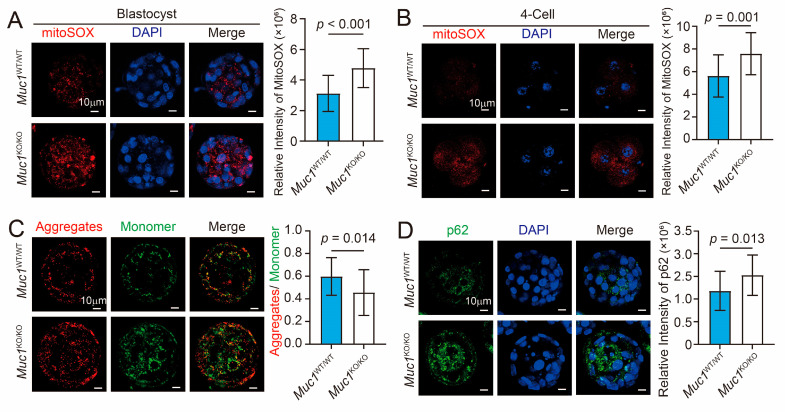
*Muc1* knockout leads to accumulation of mtROS and damaged mitochondria. (**A**) Immunofluorescence staining of MitoSOX (red) and DNA (blue) (**left**), and statistical analysis of MitoSOX fluorescence intensity (**right**) in wild-type and *Muc1* knockout groups at the blastocyst stage. Scale bar: 10 µm. Number of samples: wild-type = 22 and *Muc1* knockout = 20 (analyzed using two-tailed Student’s *t*-test). (**B**) Immunofluorescence staining of MitoSOX (red) and DNA (blue) (**left**), and statistical analysis of MitoSOX fluorescence intensity (**right**) in wild-type and *Muc1* knockout groups at the 4-cell stage. Scale bar: 10 µm. Number of samples: wild-type = 23 and *Muc1* knockout = 20 (analyzed using two-tailed Student’s *t*-test). (**C**) Immunofluorescence staining of JC-1 (mitochondrial membrane potential) (**left**), and statistical analysis of JC-1 (red-to-green ratio) (**right**) in wild-type and *Muc1* knockout groups at the blastocyst stage. Scale bar: 10 µm. Number of samples: wild-type = 23 and *Muc1* knockout = 21 (analyzed using two-tailed Student’s *t*-test). (**D**) Immunofluorescence staining of p62 (green) and DNA (blue) (**left**), and statistical analysis of p62 fluorescence intensity (**right**) in wild-type and *Muc1* knockout groups at the blastocyst stage. Scale bar: 10 µm. Number of samples: wild-type = 22 and *Muc1* knockout = 21 (analyzed using two-tailed Student’s *t*-test). Data in (**A**–**D**) are means ± SDs from at least three independent experiments.

In our previous study, MUC1 was shown to promote mitophagy in cancer cell lines [25]. Based on this, we hypothesize that MUC1 protects in vitro developmental outcomes by regulating mitophagy during early embryogenesis. To test this hypothesis, mtROS levels were assessed using MitoSOX staining. Quantitative analysis with ImageJ revealed a significant increase in mtROS levels in *Muc1*-deleted blastocysts (Figure 4A). This phenomenon was also observed in *Muc1*-null 4-cell stage embryos (Figure 4B). To determine whether the mitochondrial membrane potential was impaired during *Muc1* knockout, blastocysts were stained with JC-1. The results showed that the mitochondrial membrane potential was decreased in *Muc1* knockout blastocysts, as indicated by the weaker JC-1 fluorescence intensity (red/green ratio) (Figure 4C). This indicated that there were more damaged mitochondria when *Muc1* was knocked out. As a classic autophagic flux reporter, p62 is widely used for assessing autophagic flux in preimplantation embryos due to its high sensitivity and specificity [32]. The intracellular accumulation of p62 is inversely correlated with autophagy activity. We observed p62 accumulation in *Muc1* knockout blastocysts, indicating disrupted autophagic flux (Figure 4D). These results demonstrate that *Muc1* deletion leads to the accumulation of mtROS and damaged mitochondria, which may be associated with impaired autophagy flux.

### 3.5. Muc1 Knockout Leads to Reduction in Mitophagy

**Figure 5:**
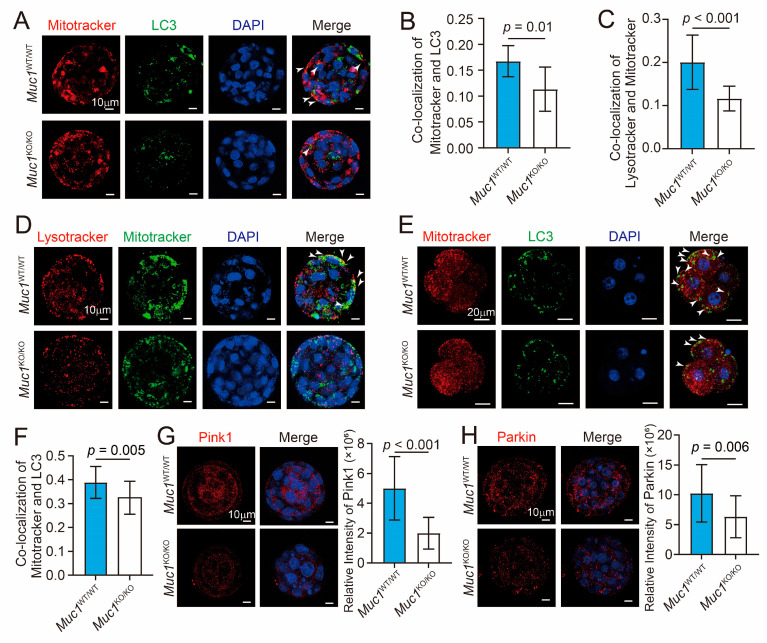
*Muc1* knockout leads to reduction in mitophagy. (**A**) Immunofluorescence staining of LC3 and MitoTracker in wild-type and *Muc1* knockout groups at the blastocyst stage. Blue, DNA; green, LC3; red, MitoTracker. Scale bar: 10 µm. White arrows indicate the co-localization of LC3 and MitoTracker. (**B**) Pearson correlation coefficient for co-localization of LC3 and MitoTracker at the blastocyst stage. Number of samples: wild-type = 28, *Muc1* knockout = 27 (analyzed using two-tailed Student’s *t*-test). (**C**) Pearson correlation coefficient for co-localization of LysoTracker and MitoTracker at the blastocyst stage. Number of samples: wild-type = 22 and *Muc1* knockout = 21 (analyzed using two-tailed Student’s *t*-test). (**D**) Immunofluorescence staining of LysoTracker and MitoTracker in wild-type and *Muc1* knockout groups at the blastocyst stage. Blue, DNA; green, MitoTracker; red, LysoTracker. Scale bar: 10 µm. White arrows indicate co-localization of LysoTracker and MitoTracker. (**E**) Immunofluorescence staining of LC3 and MitoTracker in wild-type and *Muc1* knockout groups at the 4-cell stage. Blue, DNA; green, LC3; red, MitoTracker. Scale bar: 20 µm. White arrows indicate co-localization of LC3 and MitoTracker. (**F**) Pearson correlation coefficient for co-localization of LC3 and MitoTracker at the 4-cell stage. Number of samples: wild-type = 21, *Muc1* knockout = 20 (analyzed using two-tailed Student’s *t*-test). (**G**) Immunofluorescence staining of PINK1 (red) and DNA (blue) (**left**), and statistical analysis of PINK1 fluorescence intensity (**right**) in wild-type and *Muc1* knockout groups at the blastocyst stage. Scale bar: 10 µm. Number of samples: wild-type = 21 and *Muc1* knockout = 20 (analyzed using two-tailed Student’s *t*-test). (**H**) Immunofluorescence staining of Parkin (red) and DNA (blue) (**left**), and statistical analysis of Parkin fluorescence intensity (**right**) in wild-type and *Muc1* knockout groups at the blastocyst stage. Scale bar: 10 µm. Number of samples: wild-type = 20 and *Muc1* knockout = 20 (analyzed using two-tailed Student’s *t*-test). Data in (**B**,**C**,**F**–**H**) are means ± SDs from at least three independent experiments.

Given that MUC1 can promote mitophagy in breast cancer cell lines [33], the mitophagy level was further evaluated through co-localization of LC3 and MitoTracker. MitoTracker is a class of lipophilic cationic fluorescent probes designed for specific labeling of mitochondria in live cells [34]. LC3 is anchored to the autophagosome membrane, so the colocalization of LC3 and MitoTracker directly reflects mitophagy activity [35]. Markedly diminished co-localization of LC3 and MitoTracker was observed in *Muc1* knockout blastocysts (Figure 5A,B), suggesting attenuated mitophagy following *Muc1* knockout. This finding was supported by co-localization of LysoTracker and MitoTracker (Figure 5C,D). The fluorophore of LysoTracker only emits strong fluorescence when activated by the acidic lysosomal microenvironment, achieving specific labeling of intact lysosomes in living cells [36]. The degree of co-localization of LysoTracker and MitoTracker directly reflects the efficiency of lysosomal degradation of mitochondria. Decreased co-localization of LC3 and MitoTracker was observed in *Muc1* knockout embryos as early as the 4-cell stage (Figure 5E,F). The mechanism study indicated that the protein expression levels of PINK1 and Parkin were significantly reduced in *Muc1* knockout blastocysts (Figure 5G,H). These results confirm that *Muc1* deletion leads to diminished mitophagy, which is regulated by the PINK1/Parkin pathway.

### 3.6. Low-Dose CCCP Treatment Rescues Impaired Mitophagy and Blastocyst Formation Defects Caused by Muc1 Knockout

**Figure 6:**
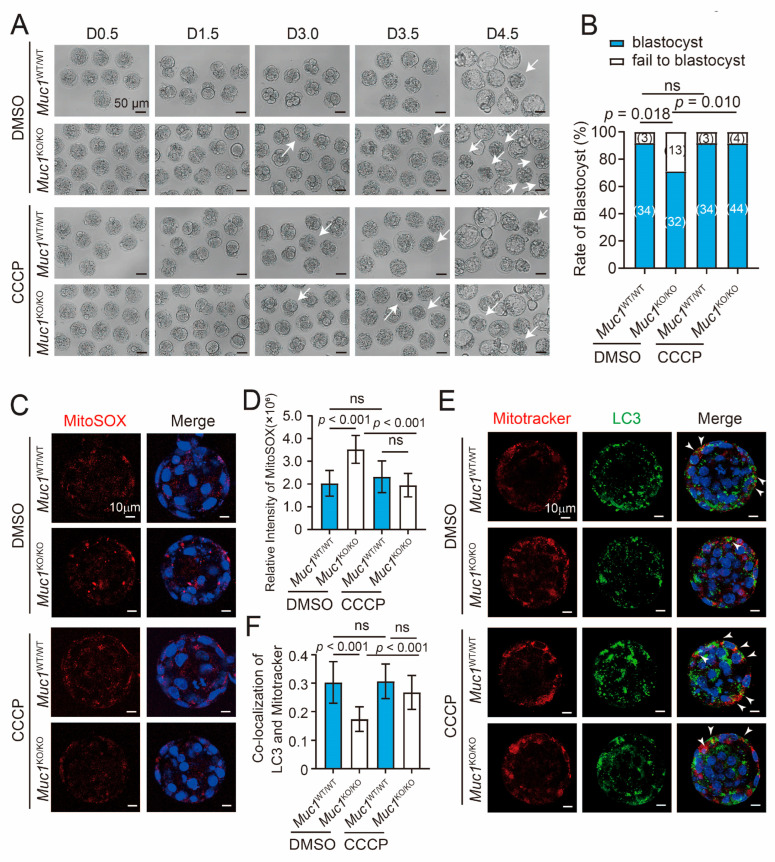
Low-dose CCCP treatment rescues impaired mitophagy and blastocyst formation defects caused by *Muc1* knockout. (**A**) Morphological comparison of D0.5, D1.5, D3.0, D3.5, and D4.5 WT, KO, WT + CCCP, and KO + CCCP embryos. Scale bar: 50 µm. White arrows indicate embryos with developmental arrest. (**B**) Blastocyst formation rates in WT, KO, WT + CCCP, and KO + CCCP groups. Number of samples analyzed in each group: WT = 37, KO = 45, WT + CCCP = 37, and KO + CCCP = 48 (analyzed using Chi-square test). (**C**) Immunofluorescence staining of MitoSOX in WT, KO, WT + CCCP, and KO + CCCP groups at the blastocyst stage. Blue, DNA; red, MitoSOX. Scale bar: 10 µm. (**D**) Relative fluorescence intensity of MitoSOX at the blastocyst stage. Number of samples analyzed in each group was as follows: WT = 20, KO = 21, WT + CCCP = 20, and KO + CCCP = 20 (analyzed using ANOVA). (**E**) Immunofluorescence staining of LC3 and MitoTracker in WT, KO, WT + CCCP, and KO + CCCP groups at the blastocyst stage. Blue, DNA; green, LC3; red, MitoTracker. Scale bar: 10 µm. White arrows indicate the co-localization of LC3 and MitoTracker. (**F**) Pearson correlation coefficient for co-localization of LC3 and MitoTracker at the blastocyst stage. Number of samples analyzed in each group: WT = 23, KO = 20, WT + CCCP = 22, and KO + CCCP = 20 (analyzed using ANOVA). Data in (**B**,**D**,**F**) are means ± SDs from at least three independent experiments. ns: not significant.

To determine if there is an association between mitophagy and blastocyst formation, wild-type (WT) and *Muc1* knockout (KO) zygotes derived from IVF were treated with low-dose CCCP (0.25 µM) to modulate mitophagy levels and determine whether this treatment could rescue the impairment induced by *Muc1* knockout. CCCP is a classic mitochondrial uncoupler and the most widely used pharmacological inducer of PINK1-dependent mitophagy [37]. It disrupts the mitochondrial membrane potential (ΔΨm) in a dose-dependent manner, with low concentrations specifically triggering mitophagy without obvious cytotoxicity and high concentrations inducing apoptosis [38]. After CCCP treatment, blastocyst formation was obviously restored in the *Muc1* knockout group, while no significant change was observed in the wild-type group (Figure 6A,B). Consistent with the above findings, *Muc1* knockout significantly increased mtROS levels. After CCCP treatment, mtROS levels were noticeably decreased in the *Muc1* knockout group, and were similar to those of the wild-type group (Figure 6C,D). Co-localization of LC3 and MitoTracker revealed a similar pattern. CCCP treatment distinctly rescued mitophagy in the *Muc1* knockout group (Figure 6E,F). These data indicate that stimulating mitophagy with low-dose CCCP can rescue the impaired blastocyst formation induced by *Muc1* knockout, demonstrating the protective role of MUC1 in early embryonic development through promoting mitophagy.

### 3.7. Vitamin C Treatment Rescues Abnormal Embryonic Development Induced by Muc1 Knockout by Normalizing mtROS Levels

**Figure 7:**
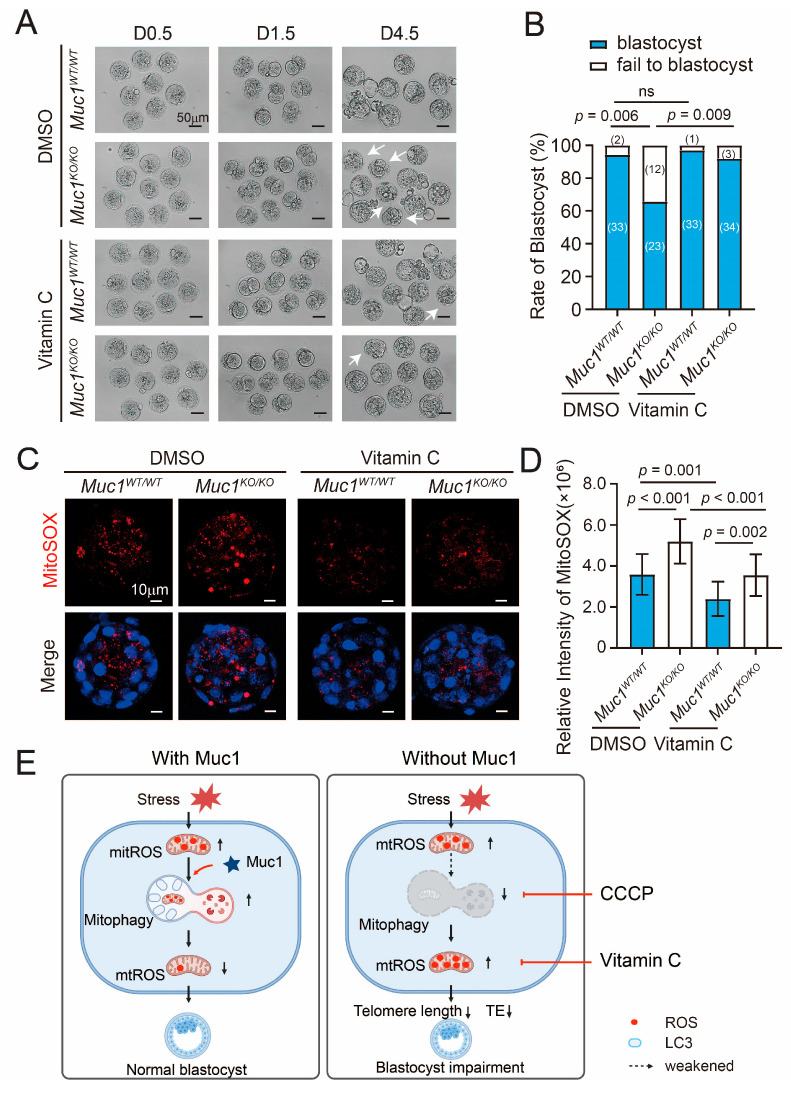
Vitamin C treatment restores abnormal embryonic development induced by *Muc1* knockout by normalizing mtROS levels. (**A**) Morphological comparison of D0.5, D1.5, and D4.5 WT, KO, WT + VC, and KO + VC embryos. Scale bar: 50 µm. White arrows indicate embryos with developmental arrest. (**B**) Blastocyst formation rates in WT, KO, WT + VC, and KO + VC groups. Number of samples analyzed in each group: WT = 35, KO = 35, WT + VC = 34, and KO + VC = 37 (analyzed using Chi-square test). (**C**) Immunofluorescence staining of MitoSOX in WT, KO, WT + VC, and KO + VC groups at the blastocyst stage. Blue, DNA; red, MitoSOX. Scale bar: 10 µm. (**D**) Relative fluorescence intensity of MitoSOX at the blastocyst stage. Number of samples: WT = 22, KO = 20, WT + VC = 20, and KO + VC = 20 (analyzed using ANOVA). (**E**) Schematic overview of the working model (created in BioRender. https://BioRender.com/z21zhau (accessed on 28th February 2026)). The dashed arrow indicates partial blockade of the process. Embryos cultured in vitro are unavoidably exposed to physical stressors, inducing the generation of mtROS. MUC1 maintains the normal progression of early embryonic development by promoting mitophagy, which suppresses mtROS accumulation. *Muc1* depletion impairs trophectoderm development and shortens telomere length. Low-dose CCCP and vitamin C treatment restore embryonic development by enhancing mitophagy and clearing mtROS, respectively. Data in (**B**,**D**) are means ± SDs from at least three independent experiments. ns: not significant.

Beyond promoting mitophagy, antioxidant responses also serve to counteract elevated ROS levels. Accordingly, the antioxidant vitamin C (VC) was supplemented into the culture medium at the documented effective concentration of 25 µg/mL [39]. The impaired blastocyst formation induced by *Muc1* knockout was restored after vitamin C treatment (Figure 7A,B). Vitamin C markedly attenuated the mtROS surge caused by *Muc1* knockout (Figure 7C,D). These findings highlight the efficacy of vitamin C in rescuing the adverse effects associated with *Muc1* insufficiency by eliminating mtROS.

## 4. Discussion

This study clarifies the crucial role of MUC1 in mouse early embryonic development. MUC1 decreases mtROS by promoting mitophagy in early embryonic development in vitro (Figure 7E), suggesting a protective function of MUC1. Additionally, vitamin C could compensate for *Muc1* deficiency by eliminating mtROS. This study not only identified a new function of MUC1 during early embryonic development in vitro, but also revealed a novel mechanism of regulating mitophagy in early embryos, with potential applications for ART.

A previous study reported that MUC1 expression is increased during spermatogenesis, specifically in germline cells [40]. MUC1 only affects post-swim-up sperm concentration, without any impact on fertilization rate or embryo quality [40]. When human embryos are co-cultured with endometrial epithelial monolayers, MUC1 expression is depleted at and adjacent to embryo attachment sites while remaining intact in neighboring cells [24]. Additionally, insufficient expression of MUC1 in the peri-implantation endometrium is associated with unexplained recurrent pregnancy loss [22]. However, few studies have characterized the expression pattern of MUC1 in early embryonic development. We found that MUC1 was expressed in both human and mouse oocytes, displaying a diffuse pattern. In sperm, immunoreactivity was restricted to the neck region in human, whereas little expression of MUC1 was detected in mouse sperm. The expression level of MUC1 modestly increased at the 4-cell stage and progressively accumulated from the morula to the blastocyst stage, reaching a peak at the blastocyst stage, suggesting a potential role of MUC1 in early embryonic development.

We next assess the effect of MUC1 on embryonic development by using *Muc1* knockout mice. Although there were no significant differences in litter size, survival rate of pups, offspring sex ratio, or interpregnancy interval compared to wild-type mice, there was a decreasing trend in offspring survival rate. Because these data were obtained from six breeding pairs, a large sample size should be included for statistical differences. Administration of an MUC1 inhibitor during pregnancy is reported to reduce litter sizes in C57BL/6j mice, indicating that MUC1 is essential for fetal survival [41]. However, in another study, no significant alterations in survival rate or fertility were observed in whole-body *Muc1* knockout mice [42].

We also explored the function of MUC1 in early embryonic development in vitro using IVF. Cultured under low (5%) oxygen conditions, the number of embryos that reached the 4-cell and blastocyst stages was significantly reduced in *Muc1* knockout embryos, indicating its critical role in early embryonic development in vitro. A randomized controlled trial reported that human preimplantation embryos cultured under ultra-low (2%) oxygen levels exhibit significantly increased blastocyst formation rates and upregulated MUC1 expression in trophectoderm cells compared with those cultured under low (5%) oxygen conditions [43].

Another group reported that MUC1 is strongly expressed in placental trophoblast cells during early pregnancy [23], suggesting a function of MUC1 in the trophectoderm. Our data demonstrated that the developmental deficit was confined to the TE in *Muc1*-null blastocysts. To explore why MUC1 only affects the TE at the blastocyst stage, co-staining of MUC1 and OCT4 was performed, and MUC1 expression was observed both in ICM and TE. The trophectoderm is not only a key structure for embryo implantation, but also the core component for nutrient and energy acquisition during early embryogenesis. Its proliferation and differentiation require a sustained and abundant ATP supply, and mitophagy serves as a central mechanism for maintaining mitochondrial homeostasis and ensuring efficient energy metabolism. At the blastocyst stage, autophagic activity is higher in trophoblast cells, consistent with their elevated energy requirement [44]. In agreement with our findings, another group reported that treatment of embryos with 5.0 µM chloroquine (CQ) significantly reduced the total cell count, while 2.5 µM CQ selectively decreased the number of Cdx2-positive cells [44]. These findings demonstrate that trophoblast cells are more sensitive to mitophagy inhibition than the inner cell mass. Thus, we propose that MUC1 specifically affects TE development due to the different sensitivities to mtROS of trophoblast and inner cell mass cells.

Telomeres are specialized nucleoprotein complexes at chromosomal ends that protect genomic integrity by preventing end-to-end fusion and degradation of chromosomes [45]. Telomere length following frozen–thawed embryo transfer provides a novel biomarker for evaluating embryo implantation potential [46]. Furthermore, telomere length has been shown to regulate mitochondrial DNA copy number in embryos [47]. Cellular oxidative stress is an important factor contributing to telomere shortening [31]. Consistently, *Muc1* knockout blastocysts showed a significant reduction in telomere length, suggesting impaired blastocyst quality.

Compared with somatic cells, mammalian embryos are more sensitive to oxidative stress, which may be attributed to the adaptation to the relatively low-oxygen environment (1–2% O_2_) in the oviduct and uterus [48]. One study shows that mtROS levels are significantly increased in blastocysts cultured in vitro compared with in vivo [49]. Excessive accumulation of ROS during in vitro culture triggers embryonic damage or developmental arrest [50] and promotes embryo fragmentation [51]. When embryos are exposed to exogenous oxidative stress, such as high oxygen tension or physical stressors, mitochondria are among the most affected organelles. A study has demonstrated that a high-oxygen environment (20% O_2_) significantly impairs embryonic mitochondrial function, including a reduced mitochondrial membrane potential and altered mitochondrial DNA copy numbers [52]. In our study, mtROS levels increased at both the 4-cell and blastocyst stages in *Muc1* knockout mice. The weaker JC-1 fluorescence intensity indicated an increase in damaged mitochondria in *Muc1* knockout blastocysts. In addition, the concomitant p62 accumulation suggested disrupted autophagic/mitophagic flux in *Muc1* knockout blastocysts, prompting us to investigate the possible role of MUC1 in mitophagy. These findings are consistent with the pattern of MUC1 expression in 4-cell and blastocyst stages, indicating the role of MUC1 in mtROS clearance.

In response to excessive ROS production, mitophagy is activated to remove damaged mitochondria, thereby limiting mtROS production and maintaining cellular homeostasis [9]. Embryos exhibit intrinsic variability in mitophagy capacity, which contributes to their differential tolerance to genetic or environmental stresses. In *Caenorhabditis elegans*, rapid mitochondrial fragmentation occurs during the oocyte-to-zygote transition (OZT), triggering mitophagy. Mitophagy during the OZT reduces the transmission of damaged mitochondrial DNA and preserves embryonic development. Interestingly, the level of autophagy in mice is high between the 2PN and 4-cell stages [53,54] and significantly increases at the morula stage, coinciding with the initiation of cavitation and progression toward blastocyst formation. At the blastocyst stage, autophagy levels are higher in trophoblast cells, consistent with the increased energy demand required by these cells [44]. Under hypoxic conditions, autophagy occurs in extravillous trophoblasts (EVTs) and promotes placental development [55]. In this study, mitophagy levels were significantly decreased in the 4-cell and blastocyst stages in *Muc1* knockout mice.

As a mitochondrial uncoupler, CCCP dissipates the mitochondrial membrane potential (ΔΨm) [56] and prevents PINK1 import into the mitochondrial matrix, triggering its accumulation on the outer mitochondrial membrane (OMM), recruiting Parkin and initiating mitophagy [37,57]. The biological processes induced by CCCP are dose-dependent. In skeletal muscle cells, 1 µM CCCP initiates mild mitophagy without obvious cytotoxicity. However, when the CCCP concentration reaches 20 µM, apoptosis will be induced [38]. In this study, low-dose CCCP exposure (0.25 µM) increased mitophagy levels, decreased mtROS levels, and restored blastocyst formation in the *Muc1* knockout group. These results suggest that the mitophagy induced by low-dose CCCP reduces mtROS levels and rescues *Muc1*-null mediated impairment of embryonic development. However, CCCP also affects multiple cellular physiological processes, such as mitochondrial metabolism [58] and general cellular stress responses [57], which may also affect the results observed in this study.

PINK1/Parkin is the most important signaling pathway for initiating mitophagy. Peroxisome proliferator-activated receptor gamma (PPAR-γ) promotes mitophagy by upregulating Beclin1, PINK1 and Parkin, reduces ROS accumulation and enhances embryonic development in Tibetan sheep [29]. In human trophoblast cells, adenosine monophosphate-activated protein kinase (AMPK) signaling stimulates mitophagy via Parkin- and FUN14-domain containing 1 (FUNDC1)-mediated pathways [59]. In mouse embryonic stem cells (ESCs), the mitophagy receptor Bcl-2/adenovirus E1B 19 kDa interacting protein 3 (BNIP3) plays a critical role in the induction and maintenance of pluripotency by mediating mitophagy [60]. Mitophagy during the OZT in *Caenorhabditis elegans* requires the mitophagy receptor FUNDC1 but the common mitophagy regulators PINK1 and BNIP3 are not involved [61]. Here, we found that MUC1 promoted mitophagy in early embryonic development through the PINK1/Parkin pathway. Notably, CCCP treatment could rescue mitophagy in the *Muc1* knockout blastocysts. These results suggest that other mechanisms could potentially compensate for impairment of the MUC1-mediated mitophagy pathways during early embryonic development.

Supplementing embryo culture medium with antioxidants is an effective strategy for counteracting ROS accumulation during in vitro culture [62]. As a well-characterized antioxidant, vitamin C (ascorbic acid) has been reported to improve embryonic development in vitro [63,64]. Vitamin C can reduce mtROS levels by enhancing the mitochondrial membrane potential and upregulating the expression of mitochondrial function-related genes, including mitofusin 1 (MFN1) and OPA1 mitochondrial dynamin-like GTPase (OPA1), as well as tricarboxylic acid cycle-related genes such as pyruvate dehydrogenase E1 subunit alpha 1 (PDHA1) and oxoglutarate dehydrogenase (OGDH) in porcine embryos in vitro [65]. Our results showed that vitamin C supplementation reduced the elevated mtROS levels caused by *Muc1* knockout and improved the developmental competence of *Muc1*-null embryos. However, vitamin C is not an mtROS-specific antioxidant; thus, mitochondria-targeted antioxidants, such as MitoTEMPO and MitoQ, may be better for specifically clearing mtROS.

To distinguish embryo-intrinsic effects from maternal contributions, we employed an IVF-based experimental system, which partly eliminated maternal in vivo environmental influences. MUC1 functions in embryos through zygotic genome activation, not maternal storage. Maternal effect factors typically act before zygotic genome activation (ZGA) and are exclusively provided by the oocyte [66]. Our data show that MUC1 expression in embryos is zygotic genome-dependent: MUC1 protein and mRNA levels remained low from the pronuclear to 8-cell stages (pre-ZGA) and increased significantly from the morula to blastocyst stages (post-ZGA), peaking at the blastocyst stage. This expression pattern indicates that the effect of MUC1 on embryonic development primarily occurs post-ZGA rather than relying on maternal MUC1 stored in the oocytes. However, given that MUC1 is expressed in multiple tissues, including epithelial tissues and reproductive organs, the global knockout model cannot exclude that the observed phenotypes reflect maternal effects, oocyte defects, uterine environment changes, and systemic physiological changes, among others.

## 5. Conclusions

Collectively, MUC1 promotes PINK1/Parkin-induced mitophagy in early embryonic development, attenuating mtROS accumulation induced by external stressors under in vitro conditions. Our results expand the functional understanding of MUC1 by revealing its protective effect on in vitro early embryonic development and uncovering a new regulatory mechanism for mitophagy in early embryos, offering promising prospects for ART applications.

## Figures and Tables

**Table 1:** Primer sequences.

Primer		Sequence
Telomere length	mB1	Forward: GCACCTTTAATCCCAGCAC
		Reverse: TGAGACAGGGTTTCTCTGTA
	Tel	Forward: CGGTTTGTTTGGGTTTGGGTTTGGGTTTGGGTTTGGGTT
		Reverse: GCCTTACCCTTACCCTTACCCTTACCCTTACCCT
RT-qPCR	*Muc1*	Forward: AGTGCCAAGTCAATACCCTGT
		Reverse: CTGGGGTGAACTGTTACTGGA
	Actin	Forward: GCAGCTCAGTAACAGTCCGC
		Reverse: AGTGTGACGTTGACATCCGT
Genotyping	*Muc1*-WT	Forward: GGCTCCTTTCTTCCTGCTGCTA
		Reverse: GATGCTAAGGAACTGCTGGTGT
	*Muc1*-KO	Forward: GCCTTCTTGACGAGTTCTTCTG
		Reverse: TGTGACTTCACGTCAGAGGCAC

## Data Availability

The original contributions presented in this study are included in the article. Further inquiries can be directed to the corresponding authors.

## Abbreviations

The following abbreviations are used in this manuscript:

2CELL	The 2-cell stage
2PN	The pronuclear stage
4CELL	The 4-cell stage
8CELL	The 8-cell stage
AMPK	Adenosine monophosphate-activated protein kinase
ANOVA	One-way analysis of variance
ART	Assisted reproductive technology
ATAD3A	ATPase family AAA domain-containing 3A
BLASTO	The blastocyst stage
BNIP3	Bcl-2/adenovirus E1B 19 kDa interacting protein 3
BSA	Bovine serum albumin
CCCP	Carbonyl cyanide 3-chlorophenylhydrazone
CDX2	Caudal type homeobox 2
COCs	Cumulus–oocyte complexes
DAPI	4′,6-diamidino-2-phenylindole
DTT	DL-dithiothreitol
eCG	Equine chorionic gonadotropin
EDTA	Ethylenediaminetetraacetic acid
ESCs	embryonic stem cells
EVTs	Extravillous trophoblasts
FL	Fluorescence intensity
FUNDC1	FUN14-domain containing 1
HBSS	Hank’s balanced salt solution
hCG	Human chorionic gonadotrophin
HTF	Human Tubal Fluid
ICM	Inner cell mass
IF	Immunofluorescence
IVF	In vitro fertilization
KO	*Muc1* knockout
KSOM	Potassium-supplemented simplex optimized medium
LSD1	Lysine-specific demethylase 1
MFN1	Mitofusin 1
MII	Metaphase II
MORULA	The morula stage
mtDNA	Mitochondrial DNA
mtROS	Mitochondria reactive oxygen species
MUC1	Mucin 1
NCtrl	Negative control
NP-40	Nonidet P 40
Oct3/4	Octamer-binding transcription factor 3/4
OGDH	Oxoglutarate dehydrogenase
OMM	Outer mitochondrial membrane
OPA1	OPA1 mitochondrial dynamin-like GTPase
OZT	Oocyte-to-zygote transition
p62	Sequestosome 1
Parkin	Parkinson protein 2
PBS	Phosphate-buffered saline
PDHA1	Pyruvate dehydrogenase E1 subunit alpha 1
PFA	Paraformaldehyde
PINK1	Phosphatase and tensin homolog (PTEN)-induced putative kinase 1
PPAR-γ	Peroxisome proliferator-activated receptor gamma
qPCR	Quantitative PCR
ROS	Reactive oxygen species
RT-qPCR	Quantitative Reverse-Transcription PCR
TE	Trophectoderm
TFR	Total fertility rate
TL	Telomere length
Tris	2-amino-2-(hydroxymethyl)-1,3-propanediol
VC	Vitamin C
WT	Wild-type
ZGA	Zygotic genome activation
ΔΨm	Mitochondrial membrane potential
